# Treatment effects of the MARA appliance and Activator-Headgear combined with fixed appliances in Class II division 1 malocclusion patients: A retrospective longitudinal study

**DOI:** 10.1590/2177-6709.27.6.e2221174.oar

**Published:** 2023-03-27

**Authors:** Deborah Brindeiro de Araújo BRITO, Silvio Augusto BELLINI-PEREIRA, Camilla Fiedler FONÇATTI, José Fernando Castanha HENRIQUES, Guilherme JANSON

**Affiliations:** 1 Universidade de São Paulo - USP, Faculdade de Odontologia de Bauru, Departamento de Ortodontia (Bauru/SP, Brazil).

**Keywords:** Headgear, Functional, Orthodontics, corrective

## Abstract

**Introduction:**

Class II division 1 malocclusion treatment with functional devices offers acceptable results. These devices can be removable or fixed, and the essential difference between them is the need for compliance. It is clinically important to investigate if there are differences in the treatment effects of these devices that present different characteristics.

**Objective:**

This retrospective longitudinal study compared the treatment effects of Class II correction with the MARA appliance, Activator-Headgear (AcHg) combination, both followed by multibracket fixed appliances, and an untreated control group.

**Material and Methods:**

Each experimental group was composed of 18 patients, with a baseline mean age of 11.70 and 10.88 years, treated for 3.60 and 3.17 years. The control group consisted of 20 subjects with baseline mean age of 11.07 years. The groups were evaluated before (T1) and after (T2) treatment. Lateral radiographs were used to evaluate the treatment changes with treatment (T2-T1), compared to the control group. Intergroup comparisons were performed using repeated-measures analysis of variance (ANOVA), followed by Tukey’s test.

**Results:**

The AcHg group showed significantly greater maxillary growth restriction than the MARA, while the mandibular changes were due to natural growth. Both devices promoted significantly greater maxillary incisors retrusion, mandibular incisors labial inclination, and improvement of overjet and molar relationships, compared to the control.

**Conclusions:**

Both functional devices followed by multibracket appliances were effective to correct Class II malocclusion. Nonetheless, the AcHg combination presents superior skeletal effects, due to significantly greater maxillary growth restriction compared to the MARA appliance. Moreover, the appliances presented similar dentoalveolar effects.

## RESUMO

**Introdução:**

O tratamento da má oclusão de Classe II divisão 1 com dispositivos funcionais oferece resultados aceitáveis. Esses dispositivos podem ser removíveis ou fixos, e a diferença essencial entre eles é a necessidade de colaboração. É clinicamente importante investigar se existem diferenças nos efeitos do tratamento desses dispositivos que apresentam características diferentes.

**Objetivo:**

O presente estudo retrospectivo longitudinal comparou os efeitos do MARA e da combinação Aparelho Extrabucal-Ativador (AEB-At) no tratamento da Classe II, ambos seguidos por aparelho fixo, adicionando também um grupo controle não tratado.

**Métodos:**

Cada grupo experimental foi composto por 18 pacientes; respectivamente, com média de idade inicial de 11,70 e 10,88 anos, tratados por 3,60 e 3,17 anos. O grupo controle foi composto por 20 indivíduos, com idade média inicial de 11,07 anos. Os grupos foram avaliados antes (T1) e após (T2) o tratamento. Radiografias de perfil foram utilizadas para avaliar as alterações do tratamento (T2-T1), em comparação com o grupo controle. As comparações intergrupos foram realizadas por meio da análise de variância para medidas repetidas (ANOVA), seguida pelo teste de Tukey.

**Resultados:**

O AEB-At apresentou uma restrição do crescimento maxilar significativamente maior que o MARA, enquanto as alterações mandibulares foram decorrentes do crescimento natural. Ambos os dispositivos promoveram significativamente mais retrusão dos incisivos superiores, inclinação vestibular dos incisivos inferiores e melhora da sobressaliência e relação molar, em comparação com o controle.

**Conclusões:**

Ambos os dispositivos funcionais associados ao aparelho fixo foram efetivos na correção da má oclusão de Classe II. No entanto, a combinação AEB-At apresenta efeitos esqueléticos superiores, devido à restrição de crescimento maxilar significativamente maior do que com o aparelho MARA. Além disso, os aparelhos apresentaram efeitos dentoalveolares semelhantes.

## INTRODUCTION

Class II is considered one of the most frequent malocclusions in the orthodontic clinic.^1^ Associated with this notable prevalence, there is an increased influx of patients searching for orthodontic treatment to treat this condition, due to its important aesthetic effect.^2^ The orthodontist can choose from a great variety of therapeutic protocols to correct Class II cases, such as: the headgear, removable or fixed functional appliances, Class II intermaxillary elastics, or orthognathic surgery.^3^

Since the main skeletal characteristic in Class II malocclusion is mandibular retrusion, and considering that protrusion of the maxilla is not often present, an approach able to redirect and stimulate mandibular growth with or without the restriction of maxillary growth would be strongly recommended.^4^ In growing patients, an early intervention with a combination of functional and multibracket fixed appliances can yield optimal treatment outcomes for Class II malocclusion.^5^

The activator and other removable functional appliances can modify Class II relationship by the transmission of soft-tissue tension to the dentition. This growth redirection can be obtained by positioning the mandible anteriorly with the appliance.^6^ However, the use of removable devices, which depend heavily on patient compliance, is a recurrent concern of orthodontists during treatment.^6^ ^,^ ^7^

Among the fixed functional appliances, there is the Mandibular Anterior Repositioning Appliance (MARA). In MARA therapy the patient is led to position his/her mandible anteriorly at rest and during masticatory function, with the advantage of being a fixed device; therefore, requiring minimum patient compliance.^1^ ^,^ ^8^ Thus, to treat Class II basal bones discrepancies the appliances should ideally generate skeletal and dental effects depending on minimal patient compliance.

Logically, the major difference between removable and fixed functional appliances is the needed amount of patient compliance. Few studies compared these different modalities and stated that removable functional appliances may be preferred when greater skeletal effects are desired.^9^ ^,^ ^10^ On the other hand, evidence suggests that the effects of removable and fixed functional appliances are similar.^11^

A recent systematic review concluded that there is still little evidence concerning the comparison of removable and fixed functional appliances.^12^ Thus, there is a need for further studies to improve clinical decision-making about this subject. Because of this controversial scenario, this study aimed to investigate the treatment effects in Class II division 1 malocclusion patients treated either with the MARA or the Activator-Headgear (AcHg) combination, both followed by multibracket fixed appliances. These groups were compared with an untreated control group of subjects with similar malocclusion.

## MATERIAL AND METHODS

This retrospective longitudinal study was previously approved by the Ethics in Research Committee of Bauru Dentistry School, University of São Paulo (São Paulo/SP, Brazil).

Sample size calculation was based on an alpha error of 5% and a beta error of 20%. The minimum mean difference of 1.5 mm in the overjet, with a standard deviation of 1.57 mm was used, based on a previous study.^13^ The sample size calculation showed that a minimum of 18 patients were required in each group.

The sample was selected from the orthodontic files of the Bauru Dentistry School/University of São Paulo, of patients treated between 2007 to 2013. The records were assessed by two operators, and all available records were organized and selected according to an *a priori* inclusion criteria. The following eligibility criteria were applied: Presence of bilateral Class II division 1 malocclusion (minimum severity of ½ cusp molar relationship); absence of agenesis; convex facial profile; and no previous history of orthodontic treatment. Additionally, the Class II division 1 patient would only be considered eligible if an ANB angle greater than 4 degrees and overbite greater than 5 mm were present. The dentition developmental stage was not considered during patient records selection.

Exclusion criteria were applied to patients that were treated with different appliances, even when initially treated with the ones of the study; and in cases of patients with incomplete records or damaged dental casts.

Data recruitment and collection were performed by the same operators through August and September 2016. Data collected included: the clinical charts, to obtain the patients’ age, general characteristics, and detailed information regarding treatment; dental casts (Class II malocclusion severity evaluation); and cephalometric radiographs at pre- (T1) and post-treatment after the use of orthopedic and multibracket orthodontic appliances (T2).

Overall, the study sample consisted of 56 subjects (36 treated, 20 untreated) divided into three groups.

The MARA group included 18 subjects (13 male, 5 female) with initial and final ages of 11.70 ± 1.11 years and 15.30 ± 1.20 years, respectively. The subjects were treated for a mean period of 3.60 ± 0.91 years, showing an initial mean ANB angle of 5.66 ± 1.49° and an initial mean overjet of 7.45 ± 1.34mm.

**Figure 1:**
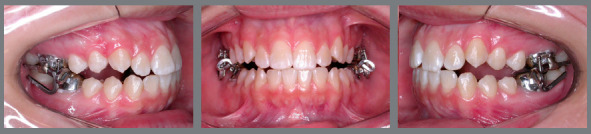
MARA appliance installed.

The MARA appliance consists of four steel crowns supported by the permanent first molars (Fig 1). These crowns include loops that connect when the patient occludes. A lingual arch and transpalatal bar were used as anchorage for the maxillary and mandibular molars, respectively.^1^ It is possible to accomplish mandibular advancement by inserting steel bands in the loops of the maxillary crowns. There are different sizes of bands (1 to 4 mm in length). In this way, advancement can be performed gradually, while the patient is able of adapt.^1^

The group treated with the AcHg combination followed by multibracket fixed appliances consisted of 18 subjects (12 male, 6 female). All patients were in the early permanent dentition. This group presented initial and final ages of 10.88 ± 0.80 years and 14.06 ± 1.35 years, respectively. The subjects were treated for a mean period of 3.17 ± 1.50 years, showing an initial ANB angle of 5.98 ± 1.64° and an initial overjet of 7.70 ± 2.30 mm.

**Figure 2:**
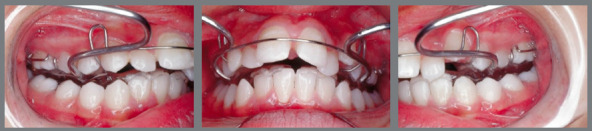
AcHg combination appliance.

The AcHg combination appliance consisted of a bimaxillary acrylic block, which included an expander screw, a 0.7-mm labial bow, and Adams clasps for retention (Fig. 2). An acrylic cape was used in the mandibular incisors to control labial tipping. The inter-occlusal acrylic area was incorporated with the Headgear bows. The construction bite was obtained guiding the mandible and incisors to an edge-to-edge relationship. The inter-occlusal space increased approximately 7 mm. Overjet greater than 7 mm was corrected with a two-step activation. Additionally, the Headgear outer bow was inclined 15º upwards from the occlusal plane, in order to exert 400g of force on each side expecting an average use of 15/h per day.^14^

The control group consisted of 20 subjects (10 male, 10 female) with untreated Class II malocclusion. This group had initial and final ages of 11.07 ±0.50 years and 15.20 ±0.93 years, respectively, and was followed-up for a mean period of 4.13 ±1.15 years. All patients were selected from the longitudinal growth study sample of the University of Toronto Burlington Growth Centre from the American Association of Orthodontics Foundation (AAOF) Craniofacial Growth Legacy Collection.

After obtaining normal anteroposterior correction, multibracket fixed appliances were installed. Aligning and leveling were performed following the sequence: 0.016-in nickel-titanium (NiTi) archwire, and 0.018-in, 0.020-in, and 0.018 x 0.025-in stainless steel archwires. Finally, the retention protocol included a Hawley plate in the maxilla and a canine-to-canine bonded retainer in the mandible. The patients were instructed to wear the Hawley retainer nights-only for 3 years, and the fixed canine-to-canine retainer for an undetermined time. Moreover, the patients were accompanied yearly to ensure the maintenance of the retainer.

After sample collection, the headfilms were digitized, traced, and analyzed with Dolphin Imaging 11.5 (Patterson Dental Supply, Chatsworth, California, USA). Image magnification factors were corrected through the software. 

**Figure 3:**
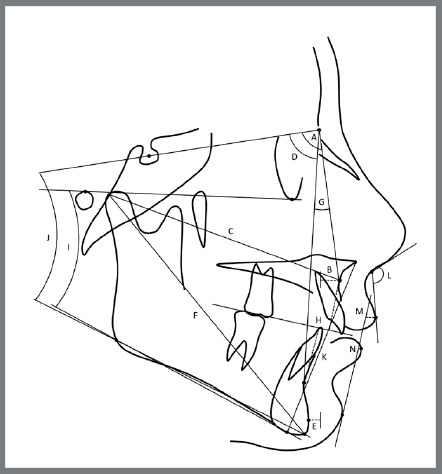
Skeletal, vertical and soft-tissue cephalometric variables: A) SNA; B) A-NPerp; C) Co-A; D) SNB; E) Pg-NPerp; F) Co-Gn; G) ANB; H) Wits; I) FMA; J) Sn.GoGn; K) LAFH; L) Nasolabial angle; M) Upper lip; N) Lower lip.

**Figure 4:**
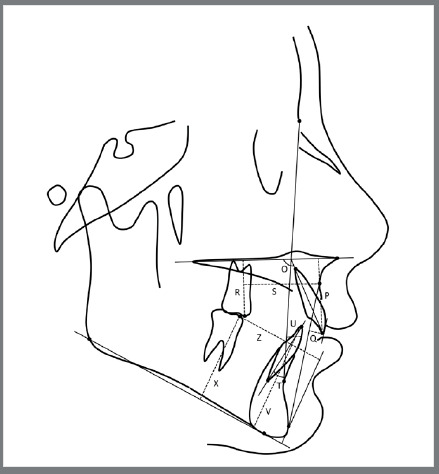
Angular and linear dentoalveolar cephalometric variables: O) Mx1.PP; P) Mx1-PP; Q) Mx1-APo; R) Mx6-PP; S) Mx6-APerp; T) Md1.NB; U) Md1-NB; V) Md1-MP; X) Md6-MP; Z) Md6-PogPerp.

Finally, an individualized cephalometric analysis was generated for each tracing (Table 1; Figs. 3 and 4). The cephalometric tracing and analysis were performed by one operator in all groups at pre- (T1) and post-treatment (T2), and the differences (T2-T1) between them were compared.

**Table 1:** Skeletal and dental cephalometric variables.

Skeletal cephalometric variables	
Maxillary component	
SNA (degrees)	Angle formed by the intersection of SN line and NA line
A-NPerp (mm)	A-point to nasion-perpendicular
Co-A (mm)	Condylion to A-point distance
Mandibular component	
SNB (degrees)	Angle formed by the intersection of SN line and NB line
Pg-NPerp (mm)	Pg-point to nasion-perpendicular
Co-Gn (mm)	Condylion to gnathion distance
Maxillomandibular relationship	
ANB (degrees)	Angle formed by the intersection of NA line and NB line
Wits (mm)	Distance between perpendicular projections of Points A and B on functional occlusal plane
Vertical component	
FMA (degrees)	Angle formed by the intersection of Frankfurt plane and Go-Me
SN.GoGn (degrees)	Angle formed by the intersection of SN line and Go-Gn
LAFH (mm)	Distance from ANS to menton
Soft-tissue component	
Nasolabial angle (degrees)	Angle formed by the Prn’-Sn line and UL-Sn’ line (Prn’ = pronasal point, Sn = subnasal point, UL = upper lip)
Upper Lip (mm)	Distance between point of the upper lip to S line (Pg’ 'point to nose)
Lower Lip (mm)	Distance between point of the lower lip to S line (Pg’ 'point to nose)
Dental cephalometric variables	
Maxillary dentoalveolar component	
Mx1.PP (degrees)	Angle formed by the maxillary incisor long axis to the palatal plane (PP)
Mx1-PP (mm)	Perpendicular distance between incisal edge of maxillary incisor and PP
Mx1-APo (mm)	Distance between incisal edge of maxillary incisor and A-Pg line
Mx6-PP (mm)	Perpendicular distance between maxillary first molar occlusal and PP
Mx6-APerp (mm)	Distance between maxillary first molar occlusal and line perpendicular to PP, tangent to A point
Mandibular dentoalveolar component	
Md1.NB (degrees)	Angle formed between the mandibular incisor long axis to NB
Md1-NB (mm)	Distance between the most anterior crown point of the mandibular incisor and NB line
Md1-MP (mm)	Perpendicular distance between incisal edge of mandibular incisor and mandibular plane
Md6-MP (mm)	Perpendicular distance between mandibular first molar occlusal and mandibular plane
Md6-PgPerp (mm)	Distance between mandibular first molar occlusal and line perpendicular to mandibular plane, tangent to Pg point
Dental relationship	
Overjet (mm)	Distance between incisal edges of maxillary and mandibular central incisors, parallel to functional occlusal plane
Overbite (mm)	Distance between incisal edges of maxillary and mandibular central incisors, perpendicular to Frankfort plane
Molar relationship (mm)	Distance between mesial points of maxillary and mandibular first molars, parallel to Frankfort plane

### ERROR STUDY

Fifty-six lateral headfilms were selected at random, re-digitized, and reassessed by the same examiner after an interval of 30 days. Dahlberg’s formula^15^ was used to estimate the random errors, and systematic errors were evaluated with dependent *t*-tests, at a significance level of *p*<0.05.^16^

### STATISTICAL ANALYSES

Initially, normal distribution was tested with Kolmogorov-Smirnov tests. Thus, all variables showed normal distribution. 

The comparability among the three groups regarding sex and initial Class II molar relationship severity was tested with Chi-square tests. Intergroup comparability regarding the initial and final ages, initial cephalometric statuses and the treatment and growth changes were performed with analysis of variance (ANOVA), followed by Tukey tests.

Statistica software (Statistica for Windows, Statsoft, Tulsa, Okla) was used to perform all statistical analyses.

## RESULTS

The random errors were within acceptable limits^14^ ^,^ ^17^ and ranged from 0.25mm (Overjet) to 1.50mm (Mx1-APo), and from 0.37º (SNB) to 1.57º (Mx1.PP). Only two (SN.GoGn and LAFH) of the 27 variables presented statistically significant systematic errors.

The MARA group showed a significantly older initial age than the headgear group, and MARA and the control group had a significantly older final age than the headgear group (Table 2). The total treatment time for the experimental groups and the follow-up period for the control group were comparable. Regarding sex and Class II molar relationship severity distributions, the groups presented great comparability.

**Table 2:** Intergroup comparability regarding initial and final ages, treatment and observation times (ANOVA followed by Tukey tests), sex distribution and severity of the initial anteroposterior relationship of the dental arches (Chi-square tests).

Variable	MARA (n=18)		AcHg (n=18)		Control (n=20)		P
	Mean	SD	Mean	SD	Mean	SD	
Initial age	11.70^A^	1.11	10.88^B^	0.8	11.07^AB^	0.5	0.012*
Final age	15.30^A^	1.2	14.06^B^	1.35	15.20^A^	0.93	0.003*
Treatment or observation time (T2-T1)	3.6	0.91	3.17	1.5	4.13	1.15	0.059
Sex distribution							
Female n(%)	5 (27.77%)		12 (66.66%)		10 (50.00%)		0.064
Male n(%)	13 (72.22%)		6 (33.33%)		10 (50.00%)		
Occlusal malocclusion severity							
½ Class II n(%)	4 (22.22%)		6 (44.44%)		6 (30.00%)		0.85
– Class II n(%)	7 (38.88%)		4 (22.22%)		6 (30.00%)		
Full-cusp Class II n(%)	7 (38.88%)		8 (44.44%)		8 (40.00%)		

Different letters indicate statistically significant differences.

*Statistically significant at *p* < 0.05.

At pretreatment, the experimental groups had significantly greater maxillary protrusion than the control (SNA: MARA = 82.23°, AcHg = 82.35°, Control = 79.78°, *p*< 0.041, Table 3). The AcHg group showed a significantly smaller maxillary length than the other two groups (Co-A = 77.63mm; *p*< 0.001). The control group presented significantly greater mandibular retrusion (Pg-NPerp = -8.56mm) than the MARA group (Pg-NPerp = -3.86mm, *p*< 0.045). The AcHg group had a significantly smaller mandibular length (Co-Gn = 102.02mm) than the control group (Co-Gn = 109.65mm, *p*< 0.000). The experimental groups showed significantly greater Class II anteroposterior discrepancies when compared to the control (ANB: MARA = 5.66°; AcHg = 5.98°; Control = 3.26°, *p*< 0.000). The control group presented a significantly greater vertical growth pattern (FMA = 25.87°; LAFH = 61.64mm) than the MARA group (FMA: 22.15°, *p*< 0.037), and greater anterior facial height than the AcHg group (LAFH = 58.17mm; *p*< 0.033). The maxillary incisors had significantly greater labial inclination (Mx1.PP), vertical dentoalveolar development (Mx1-PP), and protrusion (Mx1-APo) in the experimental groups, compared to the control. The mandibular molars were more mesially positioned in the experimental than in the control group (Md6-PgPerp: MARA = 35.68mm; AcHg = 35.09mm; Control = 39.00mm; *p*< 0.000). Likewise, the experimental groups showed significantly greater overjet than the control. The AcHg group showed a significantly smaller nasolabial angle than the control group at pretreatment (NLA: AcHg = 110.32mm; Control = 118.89mm; *p*< 0.033).

**Table 3:** Pretreatment intergroup comparability (ANOVA followed by Tukey tests).

Variable	MARA (n=18)		AcHg (n=18)		Control (n=20)		*p*
	Mean	SD	Mean	SD	Mean	SD	
Maxillary component							
SNA	82.23^A^	3.84	82.35^A^	2.91	79.78^B^	3.58	0.041*
A-NPerp	2.46^A^	3.67	1.17^A^	2.95	-2.53^B^	3.09	0.000*
Co-A	81.21^A^	3.95	77.63^B^	3.55	81.99^A^	3.70	0.001*
Mandibular component							
SNB	76.57	3.65	76.37	2.51	76.51	2.93	0.980
Pg-NPerp	-3.86^A^	5.73	-6.45^AB^	4.77	-8.56^B^	6.24	0.045*
Co-Gn	106.09^AB^	5.51	102.02^A^	4.47	109.65^B^	5.80	0.000*
Maxillomandibular relationships							
ANB	5.66^A^	1.49	5.98^A^	1.64	3.26^B^	2.55	0.000*
Wits	3.45^A^	2.57	3.94^A^	1.78	-0.14^B^	4.14	0.000*
Vertical component							
FMA	22.15^A^	4.55	25.21^AB^	3.94	25.87^B^	5.06	0.037*
SN.GoGn	31.31	5.52	32.84	4.21	32.94	4.88	0.533
LAFH	60.36^AB^	3.89	58.17^A^	3.94	61.64^B^	4.12	0.033*
Maxillary dentoalveolar component							
Mx1.PP	112.82^A^	5.02	115.48^A^	7.86	104.93^B^	7.83	0.000*
Mx1-PP	27.43^AB^	2.29	25.98^A^	2.02	28.28^B^	2.28	0.008*
Mx1-APo	8.56^A^	1.88	8.55^A^	2.35	5.87^B^	3.18	0.001*
Mx6-PP	15.91	2.39	15.29	2.03	16.05	2.43	0.568
Mx6-APerp	32.38	1.50	31.78	2.30	32.25	2.23	0.541
Mandibular dentoalveolar component							
Md1.NB	27.03	3.10	26.53	4.48	23.98	7.37	0.178
Md1-NB	4.84	1.57	4.65	1.48	3.72	2.46	0.163
Md1-MP	31.96	3.02	31.18	2.99	30.75	2.22	0.403
Md6-MP	26.25	2.28	24.67	2.42	25.98	2.10	0.090
Md6-PgPerp	35.68^A^	1.55	35.09^A^	3.51	39.00^B^	4.19	0.000*
Dentoalveolar relationship							
Overjet	7.45^A^	1.34	7.70^A^	2.30	5.12^B^	2.29	0.000*
Overbite	3.23	2.20	3.40	1.50	2.48	1.62	0.249
Molar relationship	2.59	1.27	2.50	1.66	2.75	1.98	0.103
Soft-tissue component							
Nasolabial angle	111.23^AB^	12.78	110.32^A^	11.16	118.89^B^	8.30	0.033*
Upper lip	5.28	1.66	4.76	1.96	4.04	2.02	0.134
Lower lip	3.23	2.62	3.08	2.53	1.57	2.46	0.087

Different letters indicate statistically significant differences.

*Statistically significant at *p* < 0.05.

The AcHg group showed significantly greater restriction of maxillary forward displacement, compared to the other groups (SNA = -1.87°, *p*< 0.001; Table 4). The control group presented a significantly greater increase in the maxillary effective length than the experimental groups (Co-A = 4.88mm, *p*< 0.018). At post-treatment, there was a significantly greater improvement of the Class II maxillomandibular relationship in both experimental groups, in relation to the control group (Wits = -1.49mm and -2.92mm with the MARA and AcHg, respectively, *p*< 0.000). Additionally, the Class II maxillomandibular improvement was significantly greater for the AcHg (ANB = -2.43°) compared to the MARA group (ANB = -0.99°, *p*< 0.000).

**Table 4:** Intergroup comparison of treatment and growth changes standardized to 3.17 years (T2-T1 - ANOVA followed by Tukey tests).

Variable	MARA (n=18)		AcHg (n=18)		Control (n=20)		*p*
	Mean	SD	Mean	SD	Mean	SD	
Maxillary component							
SNA	0.01^A^	2.49	-1.87^B^	2.18	0.80^A^	1.78	0.001*
A-NPerp	-0.55 ^AB^	1.96	-1.94^A^	3.30	0.38^B^	1.92	0.019*
Co-A	2.28^A^	3.66	2.28^A^	2.93	4.88^B^	2.88	0.018*
Mandibular component							
SNB	0.98	2.13	0.55	1.76	0.91	1.68	0.721
Pg-NPerp	0.63	3.81	0.36	5.27	1.06	5.11	0.902
Co-Gn	7.79	4.81	8.38	2.94	7.95	3.75	0.843
Maxillomandibular relationships							
ANB	-0.99^A^	1.51	-2.43^B^	1.65	-0.10^A^	1.27	0.000*
Wits	-1.49^A^	2.61	-2.92^A^	1.08	0.97^B^	1.54	0.000*
Vertical component							
FMA	-0.02	2.20	0.60	4.38	-0.98	3.08	0.341
SN.GoGn	-1.10	2.81	0.04^A^	3.12	-2.77	1.43	0.093
LAFH	4.86	1.92	4.47	2.39	4.31	2.11	0.209
Maxillary dentoalveolar component							
Mx1.PP	-3.34	8.07	-4.19	7.98	0.44	7.63	0.133
Mx1-PP	1.10	1.77	1.61	1.61	1.13	1.15	0.539
Mx1-APo	-2.97^A^	2.47	-2.39^A^	2.47	-0.02^B^	0.81	0.000*
Mx6-PP	2.71	1.09	2.11	2.01	2.40	1.59	0.532
Mx6-APerp	-0.84	1.97	-1.12	1.79	-1.28	1.72	0.754
Mandibular dentoalveolar component							
Md1.NB	3.27^A^	6.54	4.07^A^	4.72	-1.52^B^	4.01	0.002*
Md1-NB	1.02	1.30	1.26	1.65	0.27	1.10	0.075
Md1-MP	2.08	2.27	1.29^A^	1.83	2.87	1.64	0.066
Md6-MP	3.10	1.94	2.47	1.33	2.08	1.03	0.108
Md6-PgPerp	-1.02	1.18	0.26	2.21	-1.20	2.97	0.113
Dentoalveolar relationship							
Overjet	-4.25^A^	2.49	-4.47^A^	2.50	-0.30^B^	1.49	0.000*
Overbite	-1.41^AB^	1.86	-2.03^A^	1.49	-0.06^B^	1.58	0.001
Molar relationship	-2.64^A^	1.74	-3.10^A^	2.03	-0.50^B^	1.62	0.000*
Soft-tissue component							
Nasolabial angle	5.03	7.79	3.24	9.22	1.08	5.38	0.281
Upper lip	-1.81	1.73	-1.88	1.68	-0.95	1.30	0.136
Lower lip	-0.58	1.42	0.01	2.77	-0.65	2.01	0.545

Different letters indicate statistically significant differences.

*Statistically significant at *p* < 0.05.

The experimental groups presented significantly greater retrusion of the maxillary incisors than the control group (Mx1-APo: MARA = -2.97mm; AcHg = -2.39mm; Control = -0.02mm; *p*< 0.000). Significantly greater labial tipping was observed in the mandibular incisors in the experimental than in the control group (Md1.NB: MARA = 3.27mm; AcHg = 4.07mm; Control = -1.52mm; *p*< 0.002). Overjet and molar relationship had significantly greater improvements in the experimental than in the control group. Reductions of approximately 4.5mm in the overjet and 3mm in the molar relationship were obtained (Table 4). The AcHg had a greater improvement of the overbite than the control group.

## DISCUSSION

### SAMPLE AND METHODOLOGY

The groups tested in this study represent two distinct treatment protocols. The MARA appliance is usually installed at the permanent dentition,^1^ while the AcHg combination can be performed in the early stages of mixed dentition, ideally at the beginning of the growth spurt.^18^ Therefore, since the main focus of this study was to investigate and compare the treatment changes between these two protocols, that limitation regarding age comparability might be acceptable.

Notwithstanding, the groups presented approximately a 1-year difference in the initial and final ages (Table 2). The patients’ age could be a confounding factor in this particular comparison, overestimating the effects of one appliance. Therefore, to conduct reliable statistical comparisons, all cephalometric variables were annualized, as previously suggested.^19^ Thus, all variables of the MARA and control groups were adjusted to the time interval of the AcHg group (3.17 years). Bias due to confounding factors is common in retrospective studies, and this was an attempt to decrease the chance of introducing bias into the present study. Moreover, the samples presented great comparability regarding sex and Class II molar relationship severity distribution, which is also essential to obtain reliable results.^20^

The baseline cephalometric statuses showed statistically significant differences between the groups (Table 3). However, if the experimental groups were exclusively considered, only the initial maxillary length, represented by the Co-A variable, showed significant differences. Thus, it could be considered that experimental groups with similar initial cephalometric characteristics were compared. Finding a control that permits an exact comparison of the long-term observation between the groups would be ideal, however, very difficult to be obtained. In addition, mild differences between the experimental and control groups have been previously reported in Class II comparisons.^14^

Regarding the methodology performed, one may suggest that, in relation to the number of variables and comparisons, Bonferroni corrections^21^ should have been performed. However, the probability of detecting mild significant differences would be reduced if the correction was performed, and these small differences could be important between these two treatment protocols.

### TREATMENT EFFECTS

The three groups were compared regarding treatment outcomes and growth changes.

The AcHg showed a significantly greater maxillary growth restriction, compared to the MARA appliance. The restrictive effect of the AcHg is well established;^14^ ^,^ ^22^ however, it could be speculated that in the MARA group, the retrusion of the maxillary incisors during therapy reflected a change in the location of the A-point due to appositional changes in the alveolar area, which may have camouflaged the restrictive effect of the MARA.^23^ Similar findings were reported with other orthopedic appliances, such as the Herbst and Twin Block.^23^ Nonetheless, this topic is controversial, since previous studies found significant restrictions of maxillary growth upon treatment with the MARA appliance.^1^ ^,^ ^24^

In this study, even though the effect of the appliances seems mild regarding the SNA when the effective maxillary length is evaluated, a considerable restriction of the maxillary growth is noticed in both treated groups when compared to the control (Table 4). These findings are in accordance with other studies.^1^ ^,^ ^7^ ^,^ ^18^

The results of this work corroborate with previous studies, with no significant changes in the mandibular component between the groups^13^ ^,^ ^14^ (Table 4). Some skeletal effects on the mandible should have been expected with the MARA appliance, however, the functional appliances only induce a temporary acceleration of the mandible development, stimulating bone remodeling in the condyle and glenoid fossa while the appliance is in use.^25^ However, when the stimulus is removed, the mandibular development loses intensity gradually, until it reaches the normal values of untreated control.^25^

After treatment, the maxillomandibular relationship significantly improved in both treated groups, when compared to the control (Table 4). This improvement is a consequence of the maxillary growth restriction, associated with normal mandibular growth and significant dentoalveolar effects. Most of the researchers who have studied the MARA appliance^1^ ^,^ ^23^ ^,^ ^26^ ^,^ ^27^ and the AcHg combination,^14^ ^,^ ^28^ as well as other therapies have also reported similar findings.^7^ Nonetheless, it should be highlighted that the AcHg showed superior skeletal effects, compared to the MARA, due to its greater maxillary restrictive effect. Therefore, the AcHg combination followed by fixed appliances was more effective to improve the maxillomandibular relationship than the MARA.

Orthodontic treatment performed with AcHg and MARA followed by multibracket fixed appliances did not alter significantly the predetermined growth pattern. The changes presented in the vertical component are a probable result of normal growth. The condyle grows in a vertical direction, and the mandible presents a counterclockwise rotation in response to this growth.^29^

The maxillary dentoalveolar changes occurred mainly in the incisors (Table 4). The experimental groups presented significantly greater retrusion of the incisors than the control group. This finding corroborates with other studies and is a common effect of most functional appliances.^1^ ^,^ ^13^ ^,^ ^27^ Nonetheless, it is important to highlight that part of these significant changes derived from the multibracket fixed appliances therapy.^22^ ^,^ ^27^ Indeed, to evaluate the true effect of the AcHg combination and MARA appliances, lateral cephalograms after the orthopedic phase should have been evaluated. Thus, this limitation should be considered while interpreting the treatment effects of the appliances tested.

The maxillary molars did not present significant differences in a sagittal perspective, probably due to the use of anchorage control on experimental groups, which is in line with several studies in the literature.^13^

The experimental and control groups behaved differently regarding the mandibular incisors’ inclination. In the treated groups the incisors tipped labially, while in the control group lingual tipping was observed, therefore, demonstrating significant differences (Table 4). This significant proclination in the experimental groups has been widely reported in functional appliances therapy.^1^ ^,^ ^14^ ^,^ ^26^ Even though some lingual tipping was expected resulting from possible natural relapse and lingual torque applied during finishing, this tendency remained after treatment.^1^

Similar anteroposterior effects in the incisors and molars were observed with both appliances after the use of multibrackets (Table 4). Significant improvements in dental relationships were found. Both functional appliances presented a combination of skeletal and dental effects that in association improved the dental relationships.^1^ ^,^ ^28^

All favorable skeletal alterations were not associated with significant improvements in the soft tissue. These findings corroborate with other previous studies.^13^ ^,^ ^29^

## CONCLUSIONS

» Both treatment protocols, AcHg combination and MARA followed by multibracket fixed appliances, were effective to treat Class II division 1 malocclusion.
» The AcHg presents the advantage of promoting a significantly greater restriction of maxillary forward displacement when compared to the MARA appliance. Therefore, showing greater skeletal effects and more effectiveness in correcting the maxillomandibular relationship in Class II malocclusion patients.
» In relation to the dentoalveolar aspect, both treatment protocols showed similar effects, with a significant improvement of the molar relationships, overjet, and overbite.
